# Bullous Pilomatrixoma After COVID-19 Vaccination

**DOI:** 10.7759/cureus.32370

**Published:** 2022-12-09

**Authors:** Francisco Javier Alvarez-Rubio, Jesús Iván Martínez-Ortega, Ilse Fernández-Reyna

**Affiliations:** 1 Department of Dermatology, Instituto Dermatológico de Jalisco "Dr. José Barba Rubio", Guadalajara, MEX; 2 Department of Internal Medicine, Hospital General "Dr. Agustín O'Horán", Mérida, MEX

**Keywords:** cutaneous neoplasm, secundary anetoderma, pilomatrixoma, covid-19, bullous pilomatrixoma

## Abstract

Pilomatrixoma, or calcifying epithelioma of Malherbe, is a benign tumor with differentiation toward the hair matrix cells and is one of childhood's most common epithelial tumors. Bullous pilomatrixoma has an extremely low incidence of occurrence, usually appears in the upper extremities, and is frequently associated with trauma. We report the case of a bullous pilomatrixoma in a patient with a rapid-growing neoformation one month after receiving a coronavirus disease 2019 (COVID-19) vaccine in his left upper arm, and we discuss whether the bullous appearance is part of the biology of the tumor or a secondary anetoderma.

## Introduction

Pilomatrixoma is a benign tumor with differentiation toward the hair matrix cells that presents as a slow-growing dermal mass. Multiple variants have been reported, including bullous, anetodermal, perforating, and giant [1]. Bullous pilomatrixomas comprise 3 to 6% of all cases [2] and are usually located on the upper extremities [3]. They usually appear as solitary, flaccid, thick-walled bulla red in color, with an underlying palpable hard nodule and a larger size than a common pilomatrixoma. The treatment of choice is surgical excision with a low rate of recurrence. Some hypotheses try to explain their origin, but none are universally accepted.

Coronavirus disease 2019 (COVID-19) and its vaccines have been associated with multiple cutaneous manifestations, including local hypersensitivity reactions to generalize cutaneous vasculitis. Some case reports describe the association between bullous pilomatrixoma and influenza, hepatitis A, and COVID-19 vaccines [4]. To our knowledge, this is the second report of bullous pilomatrixoma related to COVID-19 vaccination.

## Case presentation

**Figure 1:**
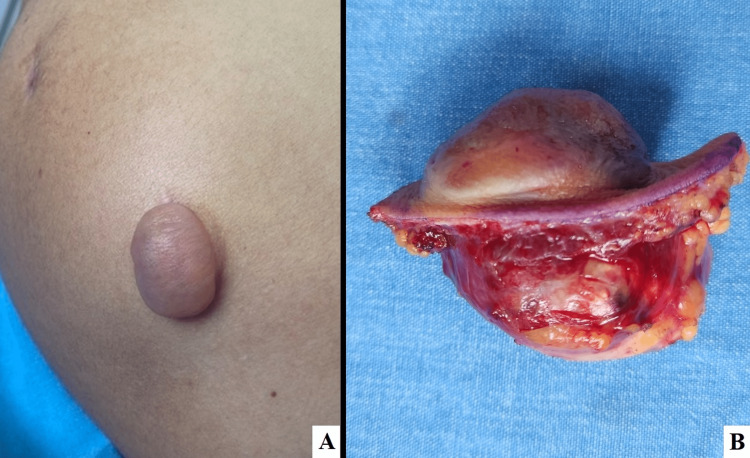
A nodule 4 cm x 2 cm x 2 cm-sized, pink-colored, bullous appearance, soft consistency, and with a hardening zone beneath (A); excisional biopsy shows a rounded tumor 4 x 4 x 4 cm-sized below the bullous appearance structure (B).

A 28-year-old male presented to our clinic complaining of a five-month-old asymptomatic tumor in the left upper arm that has rapidly grown and changed in consistency. He received the AstraZeneca COVID-19 vaccine in the left upper arm four months prior. Physical examination revealed a hemispherical nodule measuring 4 cm x 2 cm x 2 cm, pink-colored, bullous appearance, soft consistency, and with an indurated zone beneath (Figure 1).

**Figure 2:**
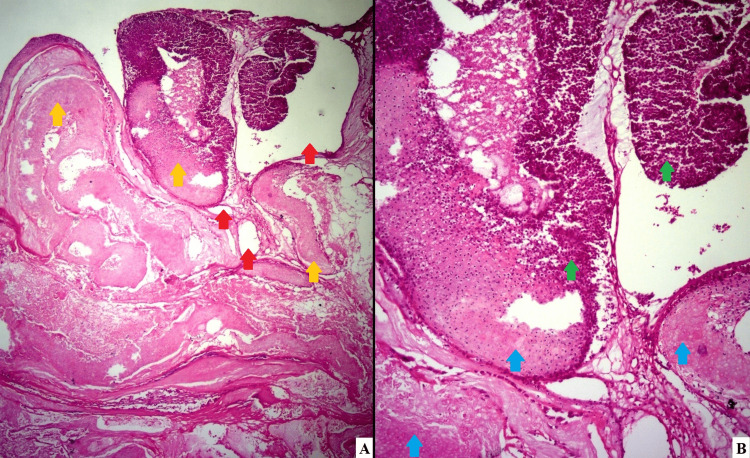
Tumor biopsy histopathology (H&E stain) (A) A lobulated dermal tumor (yellow arrows) surrounded by multiple dilated lymphatic vessels (red arrows) is seen. (B) Magnification of panel A figure shows basophilic cells (green arrow) and eosinophilic shadow cells (blue arrow) as the tumor components.

The differential diagnosis included lymphocytoma cutis, leiomyoma cutis, keloid scar, and bullous pilomatrixoma. An incisional biopsy revealed significant edema in the dermis, dilated lymphatic vessels, and an epithelial neoformation of basophilic and anucleate eosinophilic cells (ghost cells) (Figure 2), characteristics compatible with a bullous pilomatrixoma.

## Discussion

The exact etiology of pilomatrixoma is unknown. Trauma, insect bites, or surgical procedures sometimes precede the tumor [1]. Mutation in beta-catenin protein (*CTNNB1* gene), a downstream effector of the Wnt signaling pathway that influences multiple cellular processes, including hair follicle differentiation, exists in 26 to 100% of pilomatrixomas. Also, a high level of B-cell lymphoma antigen 2 (*BCL-2)* proto-oncogene is present, which is associated with the activation of neoplastic cell proliferation [5].

The etiology of the bullous appearance of some pilomatrixoma is a topic of discussion [3]. One theory holds that the growth of the tumor obstructs lymphatic vessels in the dermis, causing lymph to flow outward [1]. However, Watebe et al. and Motegi et al. found that bullous/anetodermal pilomatrixoma and the inflammatory cells around them produce elastolytic enzymes, such as metalloproteinases 9 and 12, which disrupt collagen fibers and alter lymphatic vessels [6,7]. The role of metalloproteinases in the follicular hair cycle leads us to hypothesize that it might cause the higher expression of metalloproteinases in pilomatrixoma's neoplastic cells [8].

Other tumors, such as schwannoma, xanthogranuloma, lymphoma, and plasmacytoma, have been reported to develop a bullous appearance. Several authors hypothesized that this "bullous appearance" is secondary anetoderma because of its distinctive histological features, including dermis with atrophy, edema, fragmented collagen, and disappearance of elastic fibers [3,9]. Furthermore, Li et al. did not find mutational differences between pilomatrixomas with and without bullous/anetoderma characteristics, concluding that it is an epiphenomenon [10]. Watabe et al. even indicate that perforating pilomatrixoma also presents anetodermal changes [6].

Lee et al. hypothesized that a skin tumor may be predisposed to secondary anetoderma if mechanical stress is applied. Mechanical stress on highly indurated tumors like pilomatrixoma can lead to a more significant inflammatory response with catabolic enzyme release [9]. The needle introduction in our case report may be this mechanical stress. In our opinion, most pilomatrixoma subtypes are merely different poles of a common etiopathogenic process that includes secondary anetoderma.

We propose a two-hit model in which pilomatrixoma neoplastic cells have higher expression of metalloproteinases, and mechanical stress causes a secondary inflammatory reaction, leading to an inflammatory cascade in which metalloproteinases play an essential role. Finally, secondary anetoderma develops.

On the other hand, due to the nature of the vaccine itself, namely, AZD1222/ChAdOx1 nCoV-19 Vaxzevria, Covishield (AstraZeneca), an adenovirus based-vector could cause this inflammatory cascade. Nestic et al. demonstrated that after receiving an intramuscular adenovirus-based vaccine, infected epithelial cells augmented the expression of the proinflammatory cytokines IL-6, IL-8, IL-1β, and TNF-α [11]. Whether vaccine-derived trauma or the immunogenicity/reactogenicity of the adenovirus-based vaccine is the trigger, the ongoing success of degeneration of the extracellular matrix and metalloproteinase activation would produce morphogenic tumor changes.

## Conclusions

There are a few reported cases of pilomatrixoma that develop a bullous appearance. Multiple reports of bullous pilomatrixoma are associated with trauma or vaccination, including our case report. Further research should be conducted to determine whether mechanical stress or a particular component in some vaccines is the inciting event. It is important to note that observations and studies on this matter indicate that the bullous appearance of some pilomatrixoma is, in fact, a secondary anetoderma developed on them.
