# Correction: Bullous Pilomatrixoma After COVID-19 Vaccination

**DOI:** 10.7759/cureus.c97

**Published:** 2023-01-23

**Authors:** Francisco Javier Alvarez-Rubio, Jesús Iván Martínez-Ortega, Ilse Fernández-Reyna

**Affiliations:** 1 Department of Dermatology, Instituto Dermatológico de Jalisco "Dr. José Barba Rubio", Guadalajara, MEX; 2 Department of Internal Medicine, Hospital General "Dr. Agustín O'Horán", Mérida, MEX

This article has been corrected at the request of the journal editorial department to include additional details regarding the manufacturer of the administered vaccine. In addition to including the manufacturer (AstraZeneca), the authors have provided an additional paragraph (and corresponding reference) which has been inserted at the end of the Discussion section and included here for convenience:

On the other hand, due to the nature of the vaccine itself, namely, AZD1222/ChAdOx1 nCoV-19 Vaxzevria, Covishield (AstraZeneca), an adenovirus based-vector could cause this inflammatory cascade. Nestic et al. demonstrated that after receiving an intramuscular adenovirus-based vaccine, infected epithelial cells augmented the expression of the proinflammatory cytokines IL-6, IL-8, IL-1β, and TNF-α. Whether vaccine-derived trauma or the immunogenicity/reactogenicity of the adenovirus-based vaccine is the trigger, the ongoing success of degeneration of the extracellular matrix and metalloproteinase activation would produce morphogenic tumor changes.

Reference 11: Nestić D, Božinović K, Drašković I, et al.:Human adenovirus type 26 induced IL-6 gene expression in an αvβ3 integrin- and NF-κB-dependent manner. Viruses. 2022, 14:672. 10.3390/v14040672

